# How does training in anesthesia residency shape residents’ approaches to patient care handoffs? A single-center qualitative interview study

**DOI:** 10.1186/s12909-018-1387-8

**Published:** 2018-11-20

**Authors:** Madhavi Muralidharan, Justin T. Clapp, Bridget Perrin Pulos, Sushmitha P. Diraviam, Dimitry Y. Baranov, Emily K. B. Gordon, Meghan B. Lane-Fall

**Affiliations:** 1 Department of Anesthesiology and Critical Care, Perelman School of Medicine, University of Pennsylvania, 423 Guardian Drive, 309 Blockley Hall, Philadelphia, PA 19104 USA; 2 Penn Center for Perioperative Outcomes Research and Transformation, Perelman School of Medicine, University of Pennsylvania, Philadelphia, PA USA; 3 Department of Anesthesiology and Perioperative Medicine, Mayo Clinic, Rochester, MN USA

**Keywords:** Anesthesia residents, Handoffs, Qualitative research, Graduate medical education, Patient safety, Communication

## Abstract

**Background:**

Handoffs are a complex procedure whose success relies on mutual discussion rather than simple information transfer. Particularly among trainees, handoffs present major opportunities for medical error. Previous research has explored best practices and pitfalls in general handoff education but has not discussed barriers specific to anesthesiology residents. This study characterizes the experiences of residents in anesthesiology as they learn handoff technique in order to inform strategies for teaching this important component of perioperative care.

Methods: In 2016, we conducted a semi-structured interview study of 30 anesthesia residents across all three postgraduate years at a major academic hospital. Interviews were coded by two coders using a grounded theory approach and an iterative process designed to enhance reliability and validity.

**Results:**

Residents cited lack of consistency as a major impediment to proper handoff education. They found the impact of lectures and written materials to be limited. The level of guidance and direction they received from one-to-one attendings was described as highly variable. Residents’ comfort in executing handoffs was heavily dependent on location and situation. They felt that coordination among the parties involved in the handoff was difficult to achieve, causing confusion about the importance of handoffs as well as proper protocol. Finally, residents offered opinions on when handoff education should occur during the residency and had several recommendations for its improving, including standardization of key handoff topics.

**Conclusions:**

In a single center study of anesthesiology resident handoff education, residents exhibited confusion related to a perceived disconnect between the stated importance of effective handoffs and a lack of consensus on proper handoff technique. Standardization of curriculum and framing expectations has the potential to enhance resident handoff training in academic anesthesia departments.

**Electronic supplementary material:**

The online version of this article (10.1186/s12909-018-1387-8) contains supplementary material, which is available to authorized users.

## Background

The execution of handoffs or ‘handovers’ as they are also known, is a matter of international concern. In May 2007, the World Health Organization (WHO) published a Patient Safety Solution entitled ‘Communication during Patient Hand-Overs’ due to problems with handoffs in multiple countries, including the United States, Australia, the United Kingdom of Great Britain, and Northern Ireland [1]. For the purposes of this study, we felt it appropriate to use the term ‘handoff’, rather than ‘handover,’ as this is consistent with our subjects’ understanding of the topic, though both terms are valid.

As a result of the increased focus on communication, handoff education has also become a topic of international concern [2, 3]. Handoffs have become an area of particular interest in the United States since the Accreditation Council for Graduate Medical Education (ACGME) duty hour reforms were implemented, limiting the number of hours medical trainees could work and thereby increasing the number of handoffs occurring [4]. Particularly among trainees, handoffs present a major opportunity for mistakes that compromise the quality of patient care [5]. Though the quality of handoffs has traditionally been characterized solely by the thoroughness of the information exchanged, the execution of an effective sign-out interaction is much more complex [6]. Its success relies on more than just information delivery, requiring a mutual comprehension of the salient aspects of a patient as well as a transfer of responsibility for the patient’s care [7, 8].

The complexity of the handoff process in combination with its ramifications for patient outcomes suggest the importance of studying handoffs not only in the context of clinical practice but also as a component of the residency curriculum. Anesthesiology residents, as a critical part of the perioperative team, oversee numerous handoffs over a patient’s hospital stay. The quality of these handoffs has been shown to significantly affect surgical outcomes [9, 10]. However, literature on perioperative handoffs seldom focuses on how the skill should be taught to anesthesia residents [11]. Additionally, most resident handoff education research describes the implementation and effects of initiatives to improve handoffs without significant examination of how residents experience the content or delivery of the curriculum [12].

This study sought to describe resident views on handoff education in the anesthesiology department of a major teaching hospital. Semi-structured interviews were used to assess how trainees respond to the various components of the handoff curriculum, their perceptions of its efficacy, and their ideas for how it can be improved. Such information about a critical part of the resident curriculum can help to identify and rectify gaps in training that have substantial implications for patient care.

## Methods

This study was reviewed and approved by the University of Pennsylvania Institutional Review Board (study number 822158). Written informed consent was obtained prior to resident participation. Reporting of this study and its findings were guided by the Standards for Reporting Qualitative Research [13]. We conducted semi-structured interviews of 30 anesthesiology residents at a major teaching hospital. All residents were eligible for participation, but we used purposive sampling to enroll 8–12 residents per postgraduate year, expecting differences in perspectives related to clinical experience. There were no exclusion criteria. The interview script (Additional file 1) was designed for this study by two investigators (M.B.L-F. and B.P.) and pilot tested with two residents, after which minor modifications were made. The interviews were all conducted in person, by one investigator, a resident physician at the time of the interviews (B.P.). Participants were compensated with a gift card for a local coffee vendor (value $5 USD). All interviews were recorded and professionally transcribed.

We analyzed the transcripts using a grounded theory approach [14]. Dedoose (SocioCultural Research Consultants) was used to manage coding. The primary coder (M.M.) developed a coding taxonomy through inductive examination of two randomly chosen interview transcripts. She used this codebook to code all transcripts, making iterative revisions to the codebook in order to increase the clarity and utility of codes. Next, another investigator (S.D.) double coded a randomly selected 50% of the transcripts. The two investigators regularly met during this double-coding process to compare results under the supervision of an experienced qualitative researcher (J.C.). All inconsistencies in applications of the codebook were discussed and resolved through consensus. Coding for the 50% of transcripts not double coded was subsequently revised by M.M. as appropriate to account for modifications to the codebook and how it was used.

## Results

The residents described their handoff training as occurring through both formal and informal mechanisms. The formal curriculum included events like lectures, faculty-facilitated small group simulations, and reading or multimedia materials distributed to them. Informal training included demonstration of handoffs by attending physicians, feedback from attendings during their one-to-one periods, and coaching by senior residents and nurses. The themes revealed by resident interviews are summarized in Table 1.

**Table 1:** Resident opinions on various components of handoff education, with illustrative quotes

Component	Interview findings	Illustrative quotes
Formal Curriculum	Though some residents found the lecture helpful, most residents were not significantly influenced by the lecture, either because they did not attend or because its contents were not memorable.	“At some point I think there was maybe like a lecture on this, in like grand rounds – not grand rounds, in like the resident lecture or something. But I’m not a hundred percent sure…it definitely did not stick. But I think I was there and I think it actually happened.” (CA3, M) “It’s probably just too much. It goes right over a lot of our heads – I think, at least for me. I do remember her having the lecture and talking about some of those things, but it was just like – it was just – it was a lot.” (CA1, M)
Informal Curriculum	Training from attending physicians was inconsistent in both the extent to which handoffs were covered and the content that they taught.	“But again, people’s personalities are so different as far as how they teach residents here. I think people who take it seriously will always do a good job. And if we make it an emphasis of this program and if we put the emphasis on it as being safe like we’re doing with this work, I think interest in it will grow. But I don’t know that you can necessarily bring everyone around to this kind of thinking.” (CA2, M)
Handoff Locations	Overall, residents feel more far more comfortable doing PACU handoffs compared to SICU handoffs.	“I felt more prepared to do like a standard PACU handoff. Versus, yeah, the SICU, I wasn’t quite sure how to do that. And especially we were told that there was something on Epic that we could fill out, that critical care handoff sheet. And it’s like sometimes, we do that, sometimes we don’t do that. I’m not even sure where that information goes, if people can look at that. So yeah, I didn’t feel too prepared for the SICU.” (CA1, M)
Coordinating Handoff	Residents agreed that consistently having all the involved parties present and attentive would greatly aid their learning of the handoff procedure but was very difficult to achieve.	“Probably the first step, is building the awareness amongst all of the CTICU, the surgical ICU, and the neurosurgical ICU, the nursing staff and the physicians and the midlevel providers in those places, that this is part of what we’re trying to improve upon is handoffs. And so if you teach only the anesthesia residents, it’ll I think fall flat on its face, because the anesthesia residents will show up ready to give this five minute speech, that’s a completely comprehensive signout. And there’ll be no one there to listen to it. So I think sort of setting the stage for an effective handoff is probably more important as a first step than educating about a handoff.” (CA3, M)
Handoff Template	Though many residents thought a template would help them learn to give thorough handoffs, they disagreed on the extent to which it should be enforced.	“I’m not sure quite how you would deploy it, but I think that the general answer is to develop a standardized process and then expect it to actually occur.” (CA3, M) “I think that after a while – I think [a template] might be helpful in the beginning, but after a while I think it could be – you don’t necessarily need it because you just get so use to everything that you need to hand off. But I think in the beginning it might be useful for the new CA1s coming in.” (CA1, F)
When to Teach	While some residents thought that handoffs should be taught during the one-to-one period, others felt that handoff education should occur later in the residency.	“I think that [handoffs] should be brought up during one-to-one time just because the – you have the time with an attending to actually do it and you’re usually there with another colleague. So they can definitely observe you.” (CA2, M) “I think one-to-one time, you’re so overwhelmed with the basics and you’re terrified about pushing medications and keeping your patient alive, that absorbing that information, that is an absolute, that’s like probably the worst time to teach about handoffs.” (CA2, F)

*CA#* refers to residency training year, *CA1* is Clinical Anesthesia year one, which for most residents is their second post-graduate training year after medical school, *F* female, *M* male

### Formal curriculum

Regarding the formal curriculum, several residents referenced a lecture on handoffs that was given in the beginning of their residency. However, many residents could not recall such a lecture, indicating “it either wasn’t memorable or [the residents] didn’t go.” (CA1, F) A few remembered being given reading or other online materials and attending small group sessions, but this was also highly inconsistent across the group. The inconsistent impact of these materials and sessions were attributed to the demands of residency, as “there’s so many emails and articles at the beginning” that some are inevitably overlooked. (CA2, F)

### Informal curriculum and attending instruction

The informal curriculum began with direct instruction from attending physicians during the initial shadowing period known as “one-to-one”, wherein new residents spend 2 weeks under the close tutelage of a single anesthesia attending physician, followed by another two-week period with a different attending. The levels of guidance and direction residents recalled receiving from their one-to-one attendings were highly variable. Residents cited a wide variety of styles that attendings preferred in performing handoffs and in teaching the topic. They acknowledged discrepancies between the attendings, with one saying that “it would be nice to all start from the same … point, just because one-to-one experiences can be so dramatically different.” (CA1, F) Some attendings were noted for offering little feedback or guidance about handoffs, or simply omitting the topic altogether. Residents whose one-to-one attendings explicitly covered handoffs cited variance in the content taught, with each attending having his or her “own style of what they like to do.” (CA3, F) Some had a “very systematic and algorithmic approach,” while others insisted “it doesn’t matter the order in which you do it as long as you find a way to make sure you cover it all.” (CA2, F) In contrast, many residents felt that senior residents were a strong resource for handoff training because “they have enough experience, but are still close enough to us that they know what’s necessary and what is going to be helpful.” (CA2, F)

### Handoff locations

Residents had variable comfort with handoffs depending on their location. Most residents felt well prepared for post-anesthesia care unit (PACU) handoffs, which are given exclusively to nurses. Several mentioned that the PACU nurses would “ask [them] targeted questions if [they] left things out,” which helped the residents quickly pick up on the critical components of PACU handoffs. (CA2, M) Many attributed their familiarity and relative comfort with PACU sign-outs to the fact that such sign-outs are “kind of what you do on one-to-ones” and practiced frequently during residency. (CA1 M) Most residents said that they had been to the PACU with their attending at least once during the one-to-one period. In contrast, residents felt much less prepared to deliver surgical intensive care unit (SICU) handoffs, which may involve fellows, attending physicians, interns, and nurses. Several did not have a single SICU patient during the one-to-one period, and most did not have an opportunity to observe a SICU handoff. Consequently, they described an initial confusion about how to navigate the process of handing off in the SICU; some simply “had no idea what to do when [they] got there.” (CA2, F) It was unclear whom they should approach to conduct the handoff, uncertainty compounded by time pressure to leave and start the next case. Many residents stated that they learned by “bumbling into the SICU,” or “doing and then getting yelled at and learning what not to do.” (CA3, F) Many residents felt that they would be best served by hearing from nurses directly on “what they think is important and if that matched what I give them on a frequent basis.” (CA2, F)

### Coordinating handoff delivery

Residents also felt that handoffs were difficult to learn without adequately “setting the stage for an effective handoff.” (CA3, M) They found it challenging to coordinate all the players to be present and attentive enough to deliver a thorough handoff. The uncoordinated environment detracted from the effectiveness of any handoff education that could occur, leading one resident to say that “there needs to be…equal focus on creating the right environment for the handoff…as there does on what the handoff needs to be in terms of content.” (CA3, M) An environment in which all parties are present and interested also provided a space for a junior resident “that is sort of afraid to ask” but does not want to miss any key points. (CA1, F) When asked about the ideal handoff, many residents stated that it would include the surgeon and anesthesiologist on the giving side and the physician or nurse assuming the patient’s care on the receiving side. However, they noted that it “rarely happens that way,” (CA3, M) with some residents feeling that “[they] treat handoffs very, very lightly [at this hospital].” (CA3, F)

### Handoff templates

Residents offered several suggestions for improvements that could be made to more effectively teach them proper handoff technique. A common suggestion was the development of a template or formula that could be incorporated into the handoff curriculum. One resident said that “encouraging a very systematic and thorough approach to [handoffs] from the beginning is probably the most important thing.” (CA2, F) Many residents felt that checklists would be helpful as a tool to ensure all topics are covered and serve as a tool that can be referred back to when eventually performing their own handoffs. Others mentioned that a guide in the OR might be helpful as a quick reference for the key points to be covered in a handoff. A sign-out function in the electronic medical record system was also suggested, while others liked the idea of having a tangible card or paper as a reference. Overall, residents were open to tools for standardizing high-quality handoffs, with one mentioning that he or she does not “think it’s something [residents] would say is extraneous or not useful.” (CA2, M) Another resident recalled adopting an acronym from “a buddy that goes to school…at [another institution]” that he or she felt “pretty much covered all the important things.” (CA1, M).

### When to teach handoffs

Residents also had recommendations for the ideal time to teach handoffs, though there was disagreement in this area. Many residents felt that there was not “a better time to learn anything than on one-to-ones. Literally everything [they knew] about anesthesia was modeled either directly, explicitly, or indirectly on one-to-ones…like ducklings, it’s when [they’re] imprinting.” (CA2, M) Others disagreed and felt that a time after the busy one-to-one period would be better for handoff education. These residents felt that during one-to-ones, residents are “so overwhelmed with the basics…that’s like probably the worst time to teach about handoffs.” (CA2, F) They also felt that the information might not be relevant to new residents who are “not quite sure how to employ [it].” (CA1, M) Most residents believed that continuing education on handoffs throughout the residency would be appropriate.

## Discussion

Existing literature shows that there is a lack of consensus about handoff best practices and, as a result, little agreement on the best way to teach young physicians about handoff technique [3, 15–18]. Our data suggest that this inconsistency takes many forms among anesthesiologists at a major academic hospital and constitutes a substantial impediment to properly educating residents in handoffs. Via both formal and informal curricula, residents had widely varying experiences in learning about handoffs from lectures and from attendings during one-to-ones. They also noted a much lower level of comfort and preparedness handing off in the SICU compared to the PACU. They felt that coordination among various parties involved in the handoff and consensus about its significance were difficult to achieve, making them uncertain about the importance of handoffs and about how they should be executed. Residents had several recommendations for lessening this confusion, including methods for standardizing handoff protocol as well as opinions about the best time to teach handoffs.

### Use of lectures

Previous work has shown that using lectures alone to teach handoffs to residents may be inadequate to communicate their importance and proper technique [11]. This is consistent with our data on the formal handoff curriculum, which show that most residents were not significantly influenced by the lecture, either because they did not attend or because its contents were not memorable. We suggest that this may be due to the overwhelming amount of information that residents receive in the beginning of their residency period as well as a lack of context for understanding the information conveyed. With so many new skills to master, a handoff lecture, as a less interactive way to deliver information, could easily be forgotten. Additionally, situations that might be used to teach handoffs in a lecture may have little meaning to first-year residents who have not had many cases yet, and therefore didactic material may be hard for them to retain.

### Informal curriculum

With regard to the informal curriculum, studies have found that few residents report having been observed and given feedback on handoffs by an attending physician [19], a trend that is also reflected in our data. Many of the residents we interviewed described a wide range in attending attitudes toward handoffs, a notion that corroborates findings from Lane-Fall et al.’s interview study of intensivists [20]. Residents often noted that certain attendings find both the practice of handoffs and handoff pedagogy of great value, while others do not believe handoffs are of much consequence relative to other skills anesthesiologists must know and deliver quite abbreviated handoffs themselves. However, many residents felt that senior residents were consistently helpful in learning about handoffs for two reasons. First, in approaching a senior resident, junior residents likely do not have to worry as much about the professional hierarchy and can ask questions without fear of being perceived as incompetent by someone who will be evaluating them. Second, many residents felt that senior residents were likely to probe and ask questions if they delivered handoffs that were incomplete or inadequate in some way, thereby offering them a learning experience.

### Role of handoff environment

When attempting to practice handoff skills after the one-to-one training period, residents found the difficulty of coordinating the handoff circumstances to be an impediment to learning the technique, a notion also echoed in previous research. Handoffs can occur amidst hectic and rushed environments that are less than ideal for a thorough exchange of information [21]. Busy nurses and physicians often find themselves hurried, distracted, or interrupted when trying to deliver handoffs, all of which constitute barriers to an interaction in which all parties are equally engaged [22]. Unfocused handoffs result from the lack of a concerted effort by surrounding players to prioritize the process over ambient distractions. Residents may interpret this lack of attention as a lack of importance placed on the information that they are to deliver about the patient and thus feel compelled to abbreviate it. This may lead to residents shortening their handoffs despite their better judgment so they can avoid giving superfluous information to the receiving provider and increase the chance that the information that they do include will be truly heard.

### Handoff template

The benefits of implementing a defined handoff protocol, a suggestion made by the residents to improve both teaching and execution, are well documented in past research. Data collected from a variety of medical disciplines as well as industries including aviation and manufacturing shows that the use of checklists and other tools to standardize handoffs results in fewer errors and omissions and better outcomes for patients [23–27]. Our interviewees varied in what they felt was an appropriate degree of rigidity, with some in favor of very structured checklists and others thinking that loose guidelines that are adaptable to specific situations would be more useful. However, the residents almost universally emphasized that increased structure based on established best practices would help in teaching them about how to perform successful handoffs. The willingness of the residents to adopt templates suggests that the structure that such standardized protocols provide is important for residents trying to master a daunting task. Formulaic approaches, though they might be loosened over time, could serve as important starting points for residents who are facing unfamiliar handoff situations.

### Significance of education timing

The question of when handoff education should best be pursued during residency is understudied. Further work must be done to ascertain which time is ideal to provide an introduction or explanation of the topic as well as determine a timeline on which to implement continuing education initiatives. While the significance of handoffs in shaping patient outcomes suggests that the handoff is a fundamental skill to be learnt as soon as a resident begins treating patients [28], many of the residents in our study stated that receiving handoff instruction prior to immersion in clinical contexts was unhelpful. Additionally, while many residents felt it important to include ongoing education to refresh their handoff knowledge periodically, others believed this was unnecessary after the skill has been mastered.

### Limitations and future progress

Our study has several limitations that must be noted when considering our findings. First, the study population was limited to anesthesiology residents at one major hospital in the United States. The responses that were elicited may not reflect the views of residents at other institutions. Additionally, since our study did not include direct observation of the various components of handoff education and relies entirely on reporting from the subjects, its results are limited by recall bias. Finally, the purposive sampling method used to recruit subjects could lead to selection bias.

The most effective way to educate anesthesiology residents about handoffs remains an understudied topic. Future research should focus on how the various components of handoff education, including one-to-one training, didactic sessions, and nurse feedback can be optimally integrated and standardized to present a clear, unified message to residents not only about how handoffs are to be done, but also about their importance to patient care.

## Conclusions

In a single center study of anesthesiology resident handoff education, residents exhibited confusion related to a perceived disconnect between the stated importance of effective handoffs and a lack of consensus on proper handoff technique. Standardization of curriculum and framing expectations has the potential to enhance resident handoff training in academic anesthesia departments.

## Additional file

Additional file 1: Interview instrument. The semi-structured interview instrument designed for and used in this study. (PDF 308 kb)

## Abbreviations

ACGME: Accreditation council on graduate medical education
BP: Bridget Pulos (an author name)
JC: Justin T. Clapp (an author name)
MLF: Meghan Lane-Fall (an author name)
MM: Madhavi Muralidharan (an author name)
PACU: Post anesthesia care unit
SD: Sushmitha Diraviam (an author name)
SICU: Surgical intensive care unit
