# Supplementary material for: How does training in anesthesia residency shape residents’ approaches to patient care handoffs? A single-center qualitative interview study
Source: BMC Med Educ. 2018 Nov 20;18:271. doi: 10.1186/s12909-018-1387-8 (PMC6245869; doi:10.1186/s12909-018-1387-8)
Supplement: Supplementary file 1 — Interview instrument. The semi-structured interview instrument designed for and used in this study. (PDF 308 kb) [file 12909_2018_1387_MOESM1_ESM.pdf]

Handoff Education Survey Interview Script

Note: Main questions are numbered. Probing questions are indented and lettered.

Introductory Language:

"I'll start with some introductory language, and then proceed to the interview.

This is a study about handoffs (also known as sign-out). In this study, we are interested in your experience with the education you received in regards to handoffs during your anesthesiology residency.
You can decline to answer any question.

Questions:

1. What year are you in your residency training?
2. Please tell me about your experience with handoff education.
3. Do you recall receiving formal handoff training?
    a. Lectures?
    b. Reading material?
4. What do you recall learning about handoffs during your one-to-one training during CA1 year?
    a. Do you recall your one-to-one attendings explicitly discussing handoffs?
    b. Were you able to observe handoffs in different scenarios during your one-to-one training? (PACU, SICU, CTSICU, MICU, another provider taking over care in the evening)
5. Talk about your training regarding both giving and receiving handoffs.
6. How do you feel about the education you received regarding handoffs?
    a. Can you tell me more about that?
7. Describe your level of preparation for the handoff situations you have encountered during residency.
8. What educational experiences did you feel were helpful in preparing you to give and receive handoffs?
9. Are there aspects of handoffs that you did not feel adequately prepared for?
10. Were you ever observed giving a handoff as part of your training?
    a. Did you receive any feedback about the handoff process?
11. How do you feel about the way handoffs are carried out in practice as compared to the way you learned about handoffs?
12. What types of approaches might be helpful for teaching residents about handoffs in the future?
13. Do you have any suggestions about how to improve the quality of handoff education that we receive during residency?
    a. Is one-to-one training the best time to teach this skill set or is there another time that might be better?
    b. Have there been any changes during residency that have affected the way you give or receive handoffs?
    c. Is there a need to have ongoing education about this topic?
14. Is there a standardized process you use for handoffs?
15. Do you recall receiving handoff training during medical school or at another point in your training (intern year, different residency program)?
16. Is there anything else about your handoff education during residency that you would like to mention?
17. Do you have any feedback about the interview questions or the study in general?

(End recording)

That concludes our interview. Thank you for your time."

LANE-FALL LAB
ADVANCING PATIENT-CENTERED QUALITY CARE
